# Who would rescue the dilemma of Chinese elderly care? An evolutionary game analysis and simulation research on the formalization of the domestic service industry with subsidy policy

**DOI:** 10.3389/fpubh.2023.1111208

**Published:** 2023-03-21

**Authors:** Meina Ren, Xiaozhu Chang, Shasha Du, Linping Liu

**Affiliations:** ^1^School of Social and Behavioral Sciences, Nanjing University, Nanjing, Jiangsu, China; ^2^Department of Social and Cultural Studies, Party School of C.P.C. Jiangsu Committee, Nanjing, Jiangsu, China

**Keywords:** elderly care demands, formalization of domestic service industry, subsidy policy, evolutionary game model, numerical simulation

## Abstract

Since China entered the aging society, the surging demand for elderly care and the industrial upgrading of “silver economy” has forced the domestic service industry to face endogenous challenges. Among them, the formalization of the domestic service industry can effectively reduce the transaction costs and risks of actors, innovate the endogenous vitality of the industry, and promote the improvement of elderly care quality through a triangular employment relationship. By constructing a tripartite asymmetric evolutionary game model of clients, domestic enterprises and governmental departments, this study uses the stability theorem of differential equations to explore the influencing factors and action paths of the system's evolutionary stable strategies (ESS), and uses the research data collected from China to assign values to models for simulation analysis. This study finds that the ratio of the initial ideal strategy, the difference between profits and costs, subsidies to clients, and subsidies or punishments for breach of contract to domestic enterprises are the key factors affecting the formalization of the domestic service industry. Subsidy policy programs can be divided into long-term and periodic programs, and there are differences in the influence paths and effects of the key factors in different situations. Increasing domestic enterprises' market share with employee management systems, formulating subsidy programs for clients, and setting up evaluation and supervision mechanisms are efficient ways through which to promote the formalization of the domestic service industry in China. Subsidy policy of governmental departments should focus on improving the professional skills and quality of elderly care domestic workers, and also encourage domestic enterprises with employee management systems at the same time, to expand the scope of service beneficiaries by running nutrition restaurants in communities, cooperating with elderly care institutions, etc.

## 1. Introduction

In the background of surging aging, establishing formalized domestic service markets is considered more cost-effective, with rising population dependency ratios and increasing female employment rates (1). As a result, it has emerged as the primary coping mechanism used by nations to address rising social and economic expenses of care, as well as rising demand for elderly care (2–4). Home-based, community-based, and institutional elderly care are three major parts of China's elderly care system. Among them, home-based elderly care accounts for ~90% (5). The absolute dominant position of it is a typical feature of Chinese elderly care.

On the one hand, the “filial piety” in traditional Chinese Confucian culture is the spiritual core of Chinese elderly care. Confucian culture believes that “the body, hair and skin, all have been received from the parents” *(shen ti fa fu, shou zhi fu mu)*, and correspondingly, parents need to be taken care personally by their children with full attention. Only in this way, can they be said to have lived a wonderful life in their senior years according to Confucianism. The care of children has become a critical criterion for measuring their degree of filial piety. Therefore, the elders without kinship care are defined as being in a pitiable state. Even based on several sayings in a current Confucianism practice, they will be losers in their own lives without support for future generations. Undoubtedly, with the continuous advancement of modernization, the fast-paced work and life have made the children lose the realistic conditions for personal care. The emergence of paid domestic services has just become a compensatory strategy for personal care. And it has gradually become the mainstream of Chinese elderly care, which would balance the dilemma of the practice of “filial piety” in modern social structure.

On the other hand, intergenerational property relations solidify Chinese elderly care. Owning real estate is a key factor in measuring the interpersonal status of modern Chinese. At the same time, renting means a lack of economic capabilities and social status, and even being called “homelessness” by surrounding people. Therefore, real estates are common gifts given by Chinese parents to their children, so that it would form a bond based on economic relationship of real estates full of legal and moral meaning between them. As we can see, it is difficult to change this absolute high-proportion home-based elderly care model in a short period. Among them, paid domestic services are the main form of market-oriented home-based elderly care, and also an important way to support the current situation of Chinese elderly care. The trend of the standardized development of the domestic service industry is called the formalization of the domestic service industry. It can effectively reduce the transaction costs and risks of actors through a triangular employment relationship. Elderly care services are an important part of domestic services. Under a huge shortage of workers accompanied by a low level of service, the formalization of the domestic service industry would help improve the quality of elderly care. Furthermore, it would also promote the positive work experience for elderly care workers, and significantly rescue the urgent dilemma of Chinese elderly care.

The formalization of the domestic service industry could be seen as the premise for the improvement of elderly care services marketization. At present, there are many problems in the domestic service market in China, such as a high market share of domestic enterprises with employee management systems (6), lack of professional quality, lack of professionalism and low education levels of domestic workers (7–9), and lack of labor rights protection for domestic workers (10). Domestic workers' professional quality is low, in our case, their work experience is poor, and the inferior competition is severe; clients lack consumer rights protection, limiting their consumption options; and domestic enterprises' functions are single and lack supervision. The problems above lead to a disorder of internal development of the domestic service industry resulting in the blockage of mechanism practice, which directly affects the market's ability to meet the elderly care needs. It is imperative to seek an effective way to promote the formalization of the domestic service industry.

Policy plays an instructive role in the development of industries frequently. In recent years, Chinese governmental departments have implemented subsidy policy for domestic enterprises with employee management systems and domestic workers. It has become an important method to promote the formalization of the domestic service industry. As such, the Opinions of the General Office of the State Council on Promoting the Quality and Expanding the Capacity of Domestic Service Industry (No. 30 [2019] of the General Office of the State Council) (referred to as “36 Articles of Domestic Service”) provide subsidy methods and objectives. This document defines domestic enterprises with employee management systems, as those “enterprises that directly sign service contracts with clients, sign labor contracts or service agreements with domestic workers in accordance with the law and pay social insurance premiums. Meanwhile, enterprises arrange domestic workers to provide services to clients, pay labor wages not lower than local minimum wage to domestic workers directly or on behalf of clients, and conduct continuous trainings and management for domestic workers.”

The facts that domestic enterprises tend to have employee management systems, and clients tend to purchase domestic services through domestic enterprises, are reflections of the development of the domestic service industry toward a healthy situation. Domestic enterprises with employee management systems can adjust the effectiveness of different strategies for domestic workers and clients, through reward and punishment settings, to achieve the formalization of the industry (11).

Can subsidy policy truly promote the formalization of the domestic service industry? How can subsidies be implemented more efficiently? Because the formalization of the domestic service industry is closely related to the strategies of governmental departments, domestic enterprises, and clients, the process of formalization can be regarded as a dynamic result of game strategies with constant adjustment by various actors. Therefore, evolutionary game theory provides an efficient analytical perspective from another research logic to evaluate the current relevant policies and, moreover, beyond the previous analysis logic based only on variable interpretation.

The main issues of this paper are as follows:

(1) In the case of government subsidies and supervision, what are the key factors affecting the healthy and orderly development of the domestic service industry? How do these factors affect the evolutionary path of the system?
(2) Subsidies as the long-term or periodic methods of governmental departments, will they affect the healthy operation of the domestic service industry? If so, what are the variables that governmental departments should prioritize in different situations?
(3) Is the current subsidy policy efficient? Does it promote the labor quality and occupational health of domestic workers? Does it really improve the quality of elderly care?

## 2. Literature review

### 2.1. Formalization of the domestic service industry

The triangular employment relationship established by the transaction actors in the domestic service market is the foothold of the formalization of domestic work, which is an important prerequisite for the realization of the formalization of the industry. This triangular employment relationship refers to the tripartite relationship established among service provider organizations, domestic workers and clients (12).

The triangular employment relationship facilitates more structured employment arrangements and establishes more formal working relationships while also promoting the formalization of the domestic service industry in four ways. First, domestic enterprises sign contracts with clients to control the workload of their domestic workers and avoid additional unpaid work (13, 14). Moreover, the constraints of domestic enterprises placed on clients can effectively improve the bargaining ability of domestic workers (15). However, the effectiveness of such contracts is a matter of concern because heterogeneity exists among domestic enterprises (16). Second, the labor wages of domestic workers are paid by domestic enterprises, which is beneficial for them to avoid the occurrence of unauthorized deductions from domestic workers' paychecks by clients (17, 18). Third, domestic workers employed by domestic enterprises are more likely to resist abuse and harassment by clients because they receive support from colleagues, ask for help from domestic enterprises, continue working by changing clients, etc., (19–21). Fourth, domestic enterprises provide trainings and other services to build domestic workers' confidence in the labor market. Usually, domestic workers also expect to earn higher wages after completing the trainings from domestic enterprises (22, 23). For example, Cai et al. (24) used data from China and found that trainings increased the monthly wages of domestic workers by 11.9%.

Previous studies have paid attention to the role of domestic enterprises in promoting the formalization of the domestic service industry, such as by signing contracts and providing vocational trainings. However, researchers are usually more inclined to explore the impact of domestic enterprises on domestic workers at the micro level, to ignore the impact of those services provided by domestic enterprises on clients, and to seldom discuss the impact of relevant policies on the choices of those functions undertaken by domestic enterprises. In fact, the formalization of the domestic service industry involves multiple actors, including relevant governmental departments, domestic enterprises and clients, all of whom interact with each other.

### 2.2. Government subsidy policy and the domestic service industry

Government policies in the field of domestic services can be divided into three categories: first, employment and immigration policies directly targeting domestic workers, which are usually implemented through domestic enterprises and/or domestic workers; second, policies that indirectly affect domestic workers by reducing taxes for clients and/or issuing service vouchers; and third, no policy (25). For example, the policy orientation of Denmark, Sweden, and Finland is tax relief, while that of Belgium and Austria is to issue service vouchers. Moreover, France and Germany have both of the above-mentioned policy orientations, while the United States is a typical example of having no policy. The main purpose of such policies is to increase the number of legally protected domestic workers by changing the way domestic services are purchased, thereby creating employment opportunities for private enterprises and reducing informal employment (3, 25).

The evaluation of the effect of policy implementation considers mainly the impact on the labor market, organizational transformation and households. For example, Windebank (26) found that the positive impact of French policies on households and domestic workers was negligible, while Bailly et al. (27) argued that the policies implemented in France to achieve the goal of job creation emphasize the number of jobs at the expense of quality of care services, and the increase in the proportion of direct employment reduces the positive effect of the policy. Moreover, Jokela (28) conducted a quantitative comparative study on the relationship between paid domestic work and precarious employment in Germany, Luxembourg, Italy, Spain and the United States at the micro level, finding that although policies were different, compared with other industries, the domestic service industry was more unstable. In addition, in the United States, which has no relevant policies, and other countries with subsidy policy, there is weak supervision of the domestic service market.

Since subsidy policy is often demand-driven, its goal is to make it easier for households to hire domestic workers, and the policy beneficiaries are often first those households that are able to pay for services. While reducing the burden of housework for dual-earner households, such policy also exacerbates gender inequality in housework, shifting housework from wealthier women to less wealthy women (3, 21, 26). Based on the empirical research of Belgium, France, Switzerland and Brazil, Tomei (29) found that working conditions for domestic workers improved slightly after governments offered an incentive to increase the number of licensed domestic enterprises, but the quality of domestic services remained uneven. Jokela (25) argued that relevant policies reflected the underestimation of paid domestic work in the regulation of visa restrictions and residency requirements and that practices such as limiting hours of service and failing to distinguish between different types of domestic work exacerbated the disadvantage of domestic workers' employment insecurity. Additionally, studies by van Walsum (30), Morel (31), and Jokela (28) drew conclusions similar to those of Jokela. Shutes and Walsh (32) argued that market-oriented policies may deepen the negative effects of identity differences such as race and citizenship differences. Furthermore, Tomei (29) argued that a more standardized employment relationship weakened the bargaining power of self-employed domestic workers with their employers, making domestic workers more competitive.

Governmental departments, domestic enterprises and clients are the main actors in promoting the formalization of the domestic service industry, but the relevant literature studies mainly the role of subsidy policy in a certain aspect of the domestic service industry through quantitative research or policy analysis. There is a lack of perspectives that can integrate different actors and of mathematical models to study the stability of triangular strategy combinations.

### 2.3. Application of evolutionary game theory in government subsidy policy

Evolutionary game theory combines game theory and Darwinian biological evolution theory to compensate for the lack of time evolution inherent in previous game theory. The core of evolutionary game theory is an evolutionary stable strategy (33). The research on actors' action strategies and operation mechanisms under subsidy policy or supervision by applying evolutionary game models focused on the fields of technological innovation and drug production. By constructing evolutionary game models, it can be seen that the government's innovation input and product subsidies have different impact mechanisms in terms of their effect on cost-advantage and cost-disadvantage enterprises in terms of choosing different innovation models (34). Because subsidies can reduce costs incurred by enterprises, policy subsidies are usually beneficial for the development of industries (35, 36). However, subsidy policy is usually only a short-term regulatory measure, so subsidy withdrawal mechanisms still need to be formulated by the relevant departments (37, 38). In addition, the government's supervision of various transaction actors in the market has positive significance in terms of promoting buyers and sellers in fulfilling contracts (39–41).

Evolutionary game models are suitable for studies of actors' action strategies and operation mechanisms under subsidy policy or supervision. The implementation of subsidy policy and government supervision has positive significance in emerging industries as well as those industries that are in urgent need of formalization. Subsidy structures, subsidy intensity, punishment mechanisms, and reward and punishment settings are important variables that affect the evolutionary stability of the system, and subsidy programs planned for the long term or periodically may also affect the realization paths of policy objectives.

Previous studies have considered evolutionary game theory because the theoretical perspective lacks attention to the domestic service industry; actually, the process of the formalization of the domestic service industry can also be regarded as a dynamic result of the constant adjustment of game strategies by various actors. Therefore, this study constructs a tripartite asymmetric evolutionary game model of governmental departments, domestic enterprises and clients; incorporates government subsidies and supervision as variables in the model; and uses the stability theorem of differential equations to explore the systematic evolutionary stability strategy, influencing factors and mechanisms of the formalization of the domestic service industry.

## 3. Data resources

The data reference information used in the initial value setting part of the simulation analysis of this research was from two sources. First, a questionnaire survey was conducted in Nanjing, Guangzhou, Foshan and Wuxi by the research group of Research on the Employment Relationship of Domestic Workers in the Internet Age of School of Social and Behavioral Sciences, Nanjing University, in 2019. The survey obtained 1,007 valid questionnaires through respondent-driven sampling, and was verified by the RDS estimator and other testing methods to obtain an approximate random sample, that is, this data can be regarded as a representative sample. Second, 51 documents, including those on domestic service subsidies, were collected from the official websites of the relevant departments of provinces (including autonomous regions and municipalities directly under the Central Government) and municipal governments in Mainland China (covering 15 provinces, autonomous regions, and municipalities). The direct subsidy targets of policies are mainly domestic enterprises with employee management systems and their domestic workers. Usually, domestic workers' subsidies must be implemented by domestic enterprises with employee management systems.

## 4. Model description and construction

### 4.1. Model description

**Figure 1:**
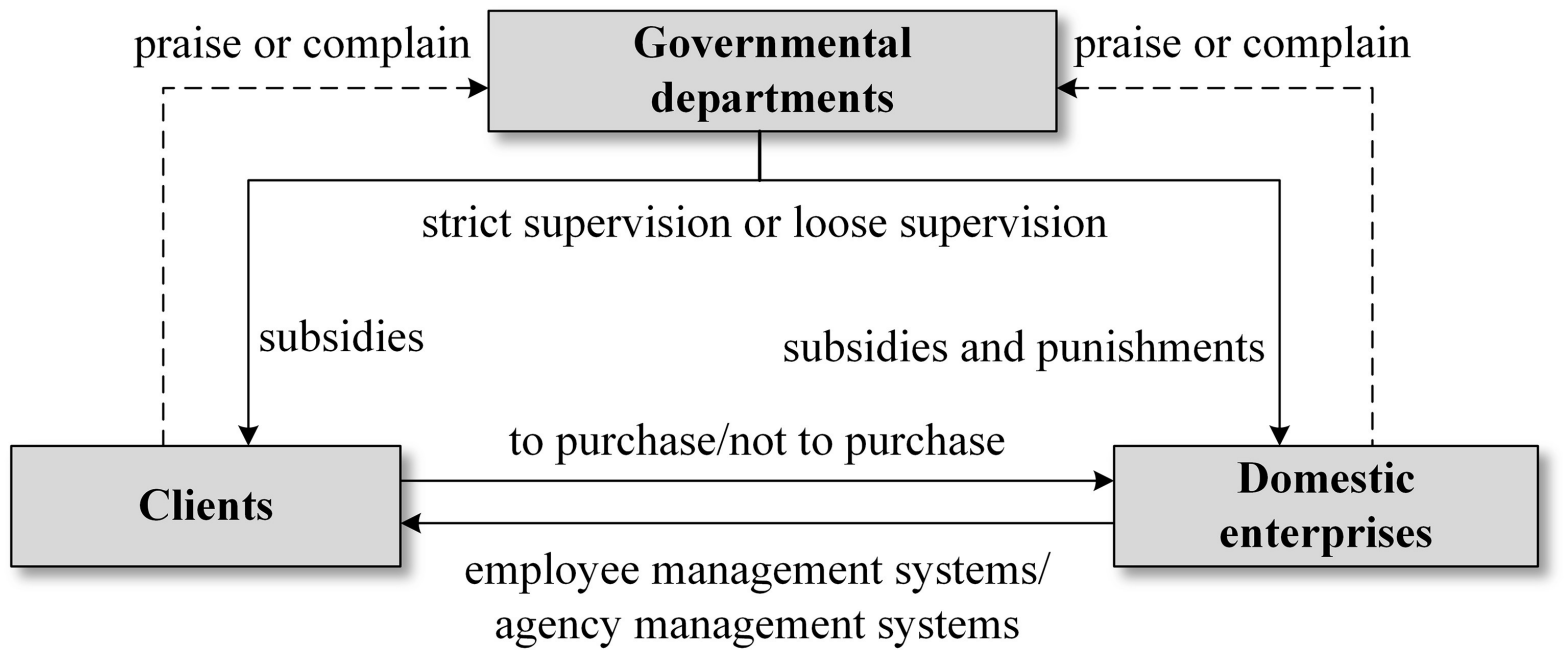
Schematic diagram of structural relationship.

Clients purchase domestic services through domestic enterprises or by hiring self-employed domestic workers. The management modes of domestic enterprises can be divided into employee management systems and agency management systems. Domestic enterprises with employee management systems sign labor contracts or service agreements with domestic workers, pay social insurance premiums, provide trainings to and pay labor wages of domestic workers, sign service contracts with clients, and establish clients rewards and feedback evaluation mechanisms. Domestic enterprises with employee management systems generally only provide order-taking and dispatching services. In addition to providing incentives through subsidy policy, governmental departments also need to set up regulatory mechanisms to restrict the behavior of actors, such as punishments for cheating in subsidies. The main goals of governmental departments are to gain a positive reputation and to improve the well-being of society; the latter includes protections for the job market, consumer rights and workers. In addition, governmental departments can also choose loose management approaches, for example, only providing market behavior regulations. Figure 1 shows the schematic diagram of structural relationship formed by the three actors.

### 4.2. Model construction

To construct evolutionary game models, the following assumptions are made: the strategic space of clients is {to purchase, not to purchase}, the proportion of those choosing to purchase domestic services is *x* (0 ≤ *x* ≤ 1), and the proportion of those choosing not to purchase domestic services is 1 − *x*. The strategic space of domestic enterprises is {employee management systems, agency management systems}, the proportion of those choosing employee management systems is *y* (0 ≤ *y* ≤ 1), and the proportion of those choosing agency management systems is 1 − *y*. The strategic space of governmental departments is {strict supervision, loose supervision}, the proportion of those with strict supervision is *z* (0 ≤ *z* ≤ 1), and the proportion of those with loose supervision is 1 − *z*. The relevant parameters are set as in Table 1.

**Table 1:** Model parameters.

**Stakeholders**	**Parameters**	**Descriptions**
Governmental departments	*B* ^*gr*^	costs of strict supervision by governmental departments
	*B* ^*gl*^	costs of loose supervision by governmental departments
	Γ	subsidy amount for domestic enterprises with employee management systems
	ϑ	subsidy intensity for domestic enterprises with employee management systems (ϑ > 0)
	Ψ	subsidy amount for clients
	φ	subsidy intensity for clients (φ > 0)
	*C* ^*cp*^	punishments for the irregular behavior of domestic enterprises with employee management systems
	θ	punishment strength for the irregular behavior of domestic enterprises with employee management systems (θ > 0)
	*D*	positive reputation and positive impact on society under strict supervision (regarded as constant)
	*N* ^*g*^	loss of governmental departments for clients choosing not to purchase domestic services from domestic enterprises
Domestic enterprises	*Q* ^*ce*^	costs of implementing employee management systems
	*Q* ^*cn*^	costs of implementing agency management systems
	*W*_*ce*_	profits from implementing employee management systems
	*W*_*cn*_	profits from implementing agency management systems
	Λ_*p*_	when governmental departments choose strict supervision, punishments for the irregular behavior of domestic enterprises with employee management systems
	*N* ^*c*^	loss of domestic enterprises for clients choosing not to purchase domestic services from domestic enterprises
Clients	*V* ^*eb*^	costs of purchasing domestic services from domestic enterprises
	*V* ^*en*^	costs of not purchasing domestic services from domestic enterprises
	*R*_*eb*_	profits from purchasing domestic services from domestic enterprises
	*R*_*en*_	profits from not purchasing domestic services from domestic enterprises
	*L*_*ec*_	when domestic enterprises choose employee management systems, rewards from purchasing domestic services from domestic enterprises
	*I* ^*b*^	loss of clients when domestic enterprises implement agency management systems

It is assumed that the sum of the profit and cost differences for different choices of clients is less than that of the rewards received from domestic enterprises, which is $R_{en}-R_{eb}+V^{eb}-V^{en}<L_{ec}$, where *V*^*eb*^ − *V*^*en*^ > 0. The sum of cost and profit differences for different choices of domestic enterprises and the rewards from purchasing domestic services for clients from domestic enterprises with employee management systems is less than the difference between subsidies and punishments imposed by governmental departments for domestic enterprises with employee management systems, which is $Q^{ce}-Q^{cn}+W_{cn}-W_{ce}+L_{ec}<\vartheta \Gamma -\theta C^{cp}$, where *Q*^*ce*^ − *Q*^*cn*^ > 0. The costs of governmental departments choosing strict supervision are higher than the costs of them choosing loose supervision, which is *B*^*gr*^ − *B*^*gl*^ > 0. The positive reputation obtained by governmental departments when they choose strict supervision is greater than their cost differences under different choices, which is *D* > *B*^*gr*^ − *B*^*gl*^. The subsidies from governmental departments to domestic enterprises with employee management systems are higher than the punishments for the irregular behavior of domestic enterprises with employee management systems, which is ϑΓ > θ*C*^*cp*^. According to the above model assumptions and parameter settings, a tripartite game payoff matrix can be obtained (see Table 2).

**Table 2:** Payoff matrix of three actors.

		**Clients**	**Governmental departments**	
			**Strict supervision**	**Loose supervision**
Domestic enterprises	Employee management systems	To purchase	$R_{eb}-V^{eb}+\varphi \Psi +L_{ec}$	$R_{eb}-V^{eb}+L_{ec}$
			$W_{ce}-Q^{ce}+\vartheta \Gamma -\theta C^{cp}-L_{ec}$	$W_{ce}-Q^{ce}-L_{ec}$
			*D* − *B*^*gr*^ − φΨ − ϑΓ + θ*C*^*cp*^	−*B*^*gl*^
		Not to purchase	$R_{en}-V^{en}$	$R_{en}-V^{en}$
			$W_{ce}-Q^{ce}+\vartheta \Gamma -\theta C^{cp}-N^{c}$	$W_{ce}-Q^{ce}-N^{c}$
		*D* − *B*^*gr*^ − ϑΓ + θ*C*^*cp*^ − *N*^*g*^	−*B*^*gl*^ − *N*^*g*^
	Agency management systems	To purchase	$R_{eb}-V^{eb}+\varphi \Psi -I^{b}$	$R_{eb}-V^{eb}-I^{b}$
			$W_{cn}-Q^{cn}-\Lambda_{p}$	$W_{cn}-Q^{cn}$
			$D-B^{gr}-\varphi \Psi +\Lambda_{p}$	−*B*^*gl*^
		Not to purchase	$R_{en}-V^{en}-I^{b}$	$R_{en}-V^{en}-I^{b}$
			$W_{cn}-Q^{cn}-\Lambda_{p}-N^{c}$	$W_{cn}-Q^{cn}-N^{c}$
			$D-B^{gr}-N^{g}+\Lambda_{p}$	−*B*^*gl*^ − *N*^*g*^

## 5. Model analysis

### 5.1. Stability analysis of clients' strategic choices

The expected profits when clients choose “to purchase” and “not to purchase” are π_*e*1_ and π_*e*2_, respectively:


(1) $$\{\begin{array}{c} \pi_{e1}=I^{b}\left(y-1\right)+\varphi \Psi z+L_{ec}y+R_{eb}-V^{eb}\\ \pi_{e2}=I^{b}\left(y-1\right)+R_{en}-V^{en} \end{array}$$

The average expected profit is $\bar{\pi_{e}}$:


(2) $$\begin{array}{l} \bar{\pi_{e}}=x\left[I^{b}\left(y-1\right)+\varphi \Psi z+L_{ec}y+R_{eb}-V^{eb}\right]+\left(1-x\right)[I^{b}\left(y-1\right)\\ \;+R_{en}-V^{en}] \end{array}$$

According to the Malthusian growth equation (42), the change rate of clients' choice “to purchase” is equal to the difference between the fitness of the strategy and average fitness. From Equations (1) and (2), the replicator dynamic equation of clients can be written as follows:


(3) $$\begin{array}{l} F\left(x\right)=\frac{dx}{dt}=x\left(\pi_{e1}-\bar{\pi_{e}}\right)\\ \;=x\left(1-x\right)\left(\varphi \Psi z+L_{ec}y-V^{eb}+V^{en}+R_{eb}-R_{en}\right) \end{array}$$

$\frac{dx}{dt}\;$ represents the change rate of the proportion of clients choosing “to purchase” with time *t* to make the strategy exist in a stable situation; it must satisfy *F*(*x*) = 0 and $\frac{\partial F\left(x\right)}{\partial x}<0$. According to Equation (3), the partial derivative of *F*(*x*) is as follows:


(4) $$\begin{array}{l} \frac{\partial F\left(x\right)}{\partial x}=\left(1-2x\right)\left(\varphi \Psi z+L_{ec}y-V^{eb}+V^{en}+R_{eb}-R_{en}\right) \end{array}$$

**Figure 2:**
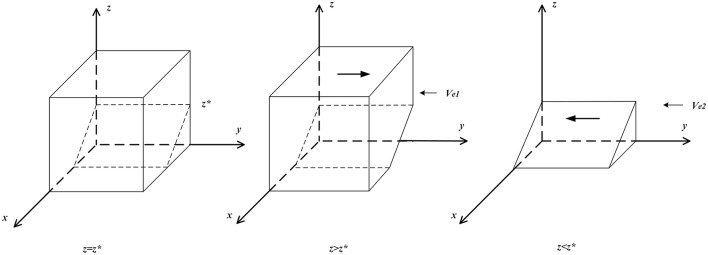
Phase diagram of clients' strategy.

(a) When $z=\frac{V^{eb}-L_{ec}y-V^{en}-R_{eb}+R_{en}}{\varphi \Psi }=z^{*}$, for any *x*, *F*(*x*) ≡ 0. At this time, any clients' strategy is a stable strategy.

(b) If *z* ≠ z^*^, then set *F*(*x*) = 0 to obtain two stable points, *x* = 0 and *x* = 1. When *z* < z^*^, ${\frac{\partial F\left(x\right)}{\partial x}|}_{x=0}<0$, $and\;{\frac{\partial F\left(x\right)}{\partial x}|}_{x=1}>0$, at this time, *x* = 0 is the evolutionary stable point; that is, when the proportion of governmental departments choosing “strict supervision” is lower than a certain degree, “not to purchase” is the optimal strategy for clients. When *z* > z^*^, $\frac{\partial F\left(x\right)}{\partial x}\;{|}_{x=1}<0$, $and\;{\frac{\partial F\left(x\right)}{\partial x}|}_{x=0}>0$, at this time, *x* = 1 is the evolutionary stable point; that is, when the proportion of governmental departments choosing “strict supervision” is higher than a certain degree, “to purchase” is the optimal strategy for clients. The related phase diagram is shown in Figure 2.

Figure 2 shows the volume *V*_*e*1_ of the probability that clients will not purchase, and the volume *V*_*e*2_ of the probability that clients will purchase, which can be calculated by the following:


(5) $$\begin{array}{l} V_{e1}=\int_{0}^{1}\int_{0}^{\frac{V^{eb}-V^{en}-R_{eb}+R_{en}}{L_{ec}}}\left[\frac{V^{eb}-V^{en}-R_{eb}+R_{en}-L_{ec}y}{\varphi \Psi }\right]dydx\\ \;=\frac{{\left(V^{eb}-V^{en}-R_{eb}+R_{en}\right)}^{2}}{2\varphi \Psi L_{ec}}V_{e2}=1-V_{e1} \end{array}$$

(6) $$\begin{array}{l} V_{e2}=1-V_{e1} \end{array}$$

**Inference I:** The probability of clients choosing “to purchase” is positively related to subsidies from governmental departments to clients and purchase incentives given to clients by domestic enterprises and is negatively related to the cost and profit differences between clients choosing “to purchase” and “not to purchase.”

**Proof:** Since $\frac{\partial V_{e2}}{\partial \varphi \Psi }>0$, $\;\frac{\partial V_{e2}}{\partial L_{ec}}>0$, $\frac{\partial V_{e2}}{\partial \Delta V_{e}}<0$, $and\;\frac{\partial V_{e2}}{\partial \Delta R_{e}}<0$, when φΨ and *L*_*ec*_ increase, the volume of *V*_*e*2_ grows, and the probability of clients choosing “to purchase” increases at this time. When Δ*V*_*e*_ and Δ*R*_*e*_ decrease, the volume of *V*_*e*2_ shrinks, and the probability of clients choosing “to purchase” decreases at this time.

From Inference I, governmental departments are shown to be able to guide clients' choices by subsidizing purchasing behavior through domestic enterprises with employee management systems.

**Inference II:** The probability of clients choosing “to purchase” increases with the increase in the probability that domestic enterprises choose “employee management systems” and governmental departments choose “strict supervision.”

**Proof:** When *z* > z^*^ or $y>\frac{V^{eb}-\varphi \Psi z-V^{en}-R_{eb}+R_{en}}{L_{ec}}$, $\frac{\partial F\left(x\right)}{\partial x}\;{|}_{x=1}<0$, *and F*(*x*) > 0, at this time, *x* = 1 is the evolutionary stable strategy. Therefore, as *y* or *z* increases, clients' stable strategy increases from *x* = 0 to *x* = 1.

From Inference II, it can be seen that when governmental departments choose “strict supervision” and when domestic enterprises implement “employee management systems,” they increase clients' purchase profits and reduce purchase costs through approaches like subsidies and incentives.

### 5.2. Stability analysis of domestic enterprises' strategic choices

The expected profits when domestic enterprises choose “employee management systems” and “agency management systems” are π_*c*1_ and π_*c*2_, respectively:


(7) $$\{\begin{array}{c} \pi_{c1}=\left(\theta C^{cp}-\vartheta \Gamma \right)z+\left(N^{c}-L_{ec}\right)x-N^{c}-Q^{ce}+W_{ce}\\ \pi_{c2}=N^{c}\left(x-1\right)-\Lambda_{p}z-Q^{cn}+W_{cn} \end{array}$$

The average expected profit is $\bar{\pi_{c}}$:


(8) $$\begin{array}{l} \bar{\pi_{c}}=y\left[\left(\theta C^{cp}-\vartheta \Gamma \right)z+\left(N^{c}-L_{ec}\right)x-N^{c}-Q^{ce}+W_{ce}\right]\\ \;+\left(1-y\right)\left[N^{c}\left(x-1\right)-\Lambda_{p}z-Q^{cn}+W_{cn}\right] \end{array}$$

From Equations (7) and (8), the replicator dynamic equation of domestic enterprises can be written as follows:


(9) $$\begin{array}{l} F\left(y\right)=\frac{dy}{dt}=y\left(\pi_{c1}-\bar{\pi_{c}}\right)=y\left(1-y\right)[\left(\vartheta \Gamma -\theta C^{cp}+\Lambda_{p}\right)z-L_{ec}x\\ \;-Q^{ce}+Q^{cn}-W_{cn}+W_{ce}] \end{array}$$

To make domestic enterprises choose “employee management systems” strategy in a stable situation, it must satisfy *F*(*y*) = 0 and $\frac{\partial F\left(y\right)}{\partial y}<0$. According to Equation (9), the partial derivative of *F*(*y*) is as follows:


(10) $$\begin{array}{l} \frac{\partial F\left(y\right)}{\partial y}=\left(1-2y\right)[\left(\vartheta \Gamma -\theta C^{cp}+\Lambda_{p}\right)z-L_{ec}x-Q^{ce}+Q^{cn}\\ \;-W_{cn}+W_{ce}] \end{array}$$

**Figure 3:**
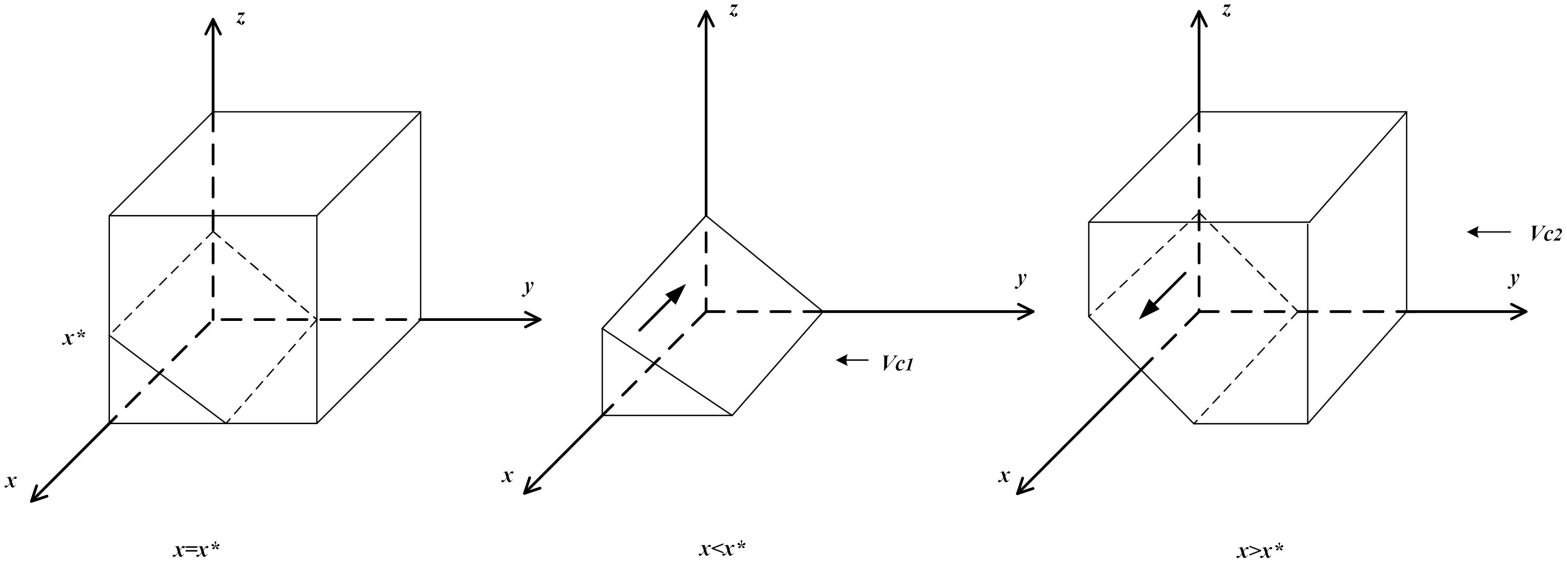
Phase diagram of domestic enterprises' strategy.

(a) When $x=\frac{\left(\vartheta \Gamma -\theta C^{cp}+\Lambda_{p}\right)z-Q^{ce}+Q^{cn}-W_{cn}+W_{ce}}{L_{ec}}=x^{*}$, for any *y*, *F*(*y*)≡0. At this time, any domestic enterprises' strategy is a stable strategy.

(b) If *x* ≠ x^*^, then set *F*(*y*) = 0 to obtain two stable points, *y* = 0 *and y* = 1. When *x* > x^*^, ${\frac{\partial F\left(y\right)}{\partial y}|}_{y=0}<0$, and ${\frac{\partial F\left(y\right)}{\partial y}|}_{y=1}>0$, then *y* = 0 is the evolutionary stable point; that is, when the probability of clients choosing “to purchase” is larger than a certain degree, “agency management systems” are the optimal strategy for domestic enterprises. When *x* < x^*^, ${\frac{\partial F\left(y\right)}{\partial y}|}_{y=1}<0$,$and\;{\frac{\partial F\left(y\right)}{\partial y}|}_{y=0}>0,\;$ then *y* = 1 is the evolutionary stable point; that is, when the probability of clients choosing “to purchase” is less than a certain degree, “employee management systems” are the optimal strategy for domestic enterprises. The related phase diagram is shown in Figure 3.

Figure 3 shows the volume *V*_*c*1_ of the probability of domestic enterprises choosing “employee management systems” and the volume *V*_*c*2_ of the probability of them choosing “agency management systems,” which can be calculated by the following:


(11) $$\begin{array}{l} V_{c1}=\int_{0}^{1}\int_{0}^{\frac{Q^{ce}+Q^{cn}-W_{cn}+W_{ce}}{\vartheta \Gamma -\theta C^{cp}+\Lambda_{p}}}\frac{\left(\vartheta \Gamma -\theta C^{cp}+\Lambda_{p}\right)z-Q^{ce}+Q^{cn}-W_{cn}+W_{ce}}{L_{ec}}dzdy\\ \;=-{\frac{\left(\vartheta \Gamma +3\theta C^{cp}+\Lambda_{p}\right)\left(Q^{ce}+Q^{cn}-W_{cn}+W_{ce}\right)}{2L_{ec}{\left(\vartheta \Gamma +\theta C^{cp}+\Lambda_{p}\right)}^{2}}}^{2} \end{array}$$

(12) $$\begin{array}{l} V_{c2}=1-V_{c1} \end{array}$$

**Inference III:** The probability of domestic enterprises choosing “employee management systems” is positively related to governmental departments' subsidies to domestic enterprises with employee management systems, rewards from domestic enterprises given to clients, and punishments for the irregular behavior of domestic enterprises with employee management systems and is negatively related to cost differences of domestic enterprises choosing “employee management systems” and “agency management systems,” profit differences between “agency management systems” and “employee management systems,” and governmental departments' punishments for the irregular behavior of domestic enterprises with employee management systems.

**Proof:** Since $\frac{\partial V_{c2}}{\partial \vartheta \Gamma }>0$, $\frac{\partial V_{c2}}{\partial L_{ec}}>0$, $\frac{\partial V_{c2}}{\partial \Lambda_{p}}>0$, $\frac{\partial V_{c2}}{\partial \Delta Q_{c}}<0$, $\frac{\partial V_{c2}}{\partial \Delta W_{c}}<0$, $\;and\;\frac{\partial V_{c2}}{\partial \theta C^{cp}}<0$, when ϑ*Γ, L*_*ec*_, and Λ_*p*_ increase, the volume of *V*_*c*1_ increases. At this time, the probability of domestic enterprises choosing “employee management systems” increases, and when Δ*Q*_*c*_, Δ*W*_*c*_, and θ*C*^*cp*^ decrease, the volume of *V*_*c*1_ decreases, and the probability of domestic enterprises choosing “employee management systems” decreases.

From Inference III, it is shown that the implementation of punishments for the irregular behavior of domestic enterprises with employee management systems by governmental departments will help domestic enterprises implement more standardized employee management systems. Moreover, punishments for the irregular behavior of domestic enterprises with employee management systems should be less than their subsidies, which is conducive to the expansion of the market share of domestic enterprises with employee management systems.

**Inference IV:** The probability of domestic enterprises choosing “employee management systems” is positively related to the probability of governmental departments choosing “strict supervision” and is negatively related to the probability of clients choosing “to purchase.”

**Proof:** When *x* < x^*^ or $z>\frac{L_{ec}x+Q^{ce}-Q^{cn}+W_{cn}-W_{ce}}{\vartheta \Gamma -\theta C^{cp}+\Lambda_{p}}$, ${\frac{\partial F\left(y\right)}{\partial y}|}_{y=1}<0$, *and F*(*y*) > 0; at this time, *y* = 1 is the evolutionary stable strategy. Therefore, as *x* decreases or *z* increases, the stable strategy of domestic enterprises increases from *y* = 0 to *y* = 1.

From Inference IV, it is shown that clients tend not to choose to purchase services from domestic enterprises and that domestic enterprises set up incentives and provide better after-sales services to attract clients. If governmental departments subsidize domestic enterprises with employee management systems and implement punishments to domestic enterprises with employee management systems for their irregular behavior, then domestic enterprises are more inclined to implement “employee management systems.”

### 5.3. Stability analysis of governmental departments' strategic choices

The expected profits when governmental departments choose “strict supervision” and “loose supervision” are π_*g*1_ and π_*g*2_, respectively:


(13) $$\{\begin{array}{c} \pi_{g1}=N^{g}\left(x-1\right)-\varphi \Psi x+\left(\theta C^{cp}-\vartheta \Gamma -\Lambda_{p}\right)y+D+B^{gr}+\Lambda_{p}\\ \pi_{g2}=N^{g}\left(x-1\right)-B^{gl} \end{array}$$

The average expected profit is $\bar{\pi_{g}}$:


(14) $$\begin{array}{l} \bar{\pi_{g}}=z\left[N^{g}\left(x-1\right)-\varphi \Psi x+\left(\theta C^{cp}-\vartheta \Gamma -\Lambda_{p}\right)y+D+B^{gr}+\Lambda_{p}\right]\\ \;+\left(1-z\right)\left[N^{g}\left(x-1\right)-B^{gl}\right] \end{array}$$

From Equations (13) and (14), the replicator dynamic equation of governmental departments can be written as follows:


(15) $$\begin{array}{l} F\left(z\right)=\frac{dz}{dt}=z\left(\pi_{g1}-\bar{\pi_{g}}\right)\\ \;=z\left(1-z\right)\left[B^{gl}-B^{gr}+\Lambda_{p}+D-\varphi \Psi x-\left(\vartheta \Gamma -\theta C^{cp}+\Lambda_{p}\right)y\right] \end{array}$$

To make governmental departments choose the strategy of “strict supervision” in a stable situation, they must satisfy $F\left(z\right)=0\;and\;\frac{\partial F\left(z\right)}{\partial z}<0$. According to Equation (9), the partial derivative of *F*(*z*) is as follows:


(16) $$\begin{array}{l} \frac{\partial F\left(z\right)}{\partial z}=\left(1-2z\right)\left[B^{gl}-B^{gr}+\Lambda_{p}+D-\varphi \Psi x-\left(\vartheta \Gamma -\theta C^{cp}+\Lambda_{p}\right)y\right] \end{array}$$

**Figure 4:**
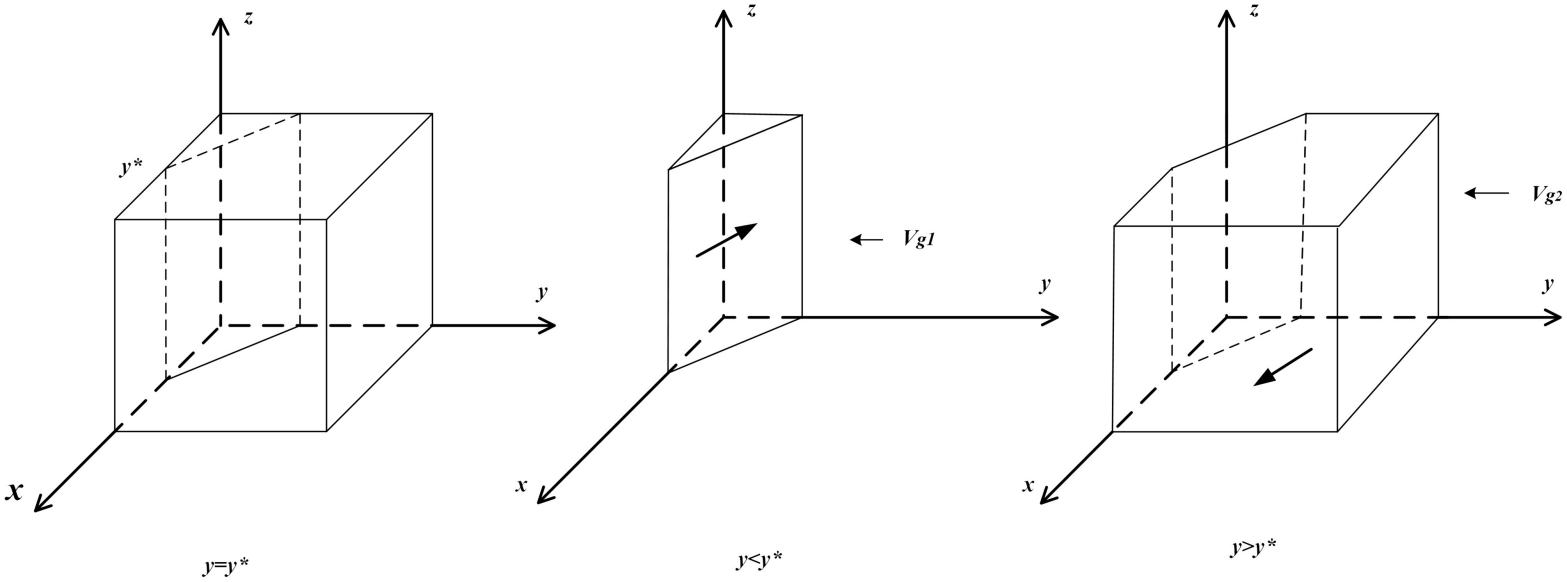
Phase diagram of governmental departments' strategy.

(a) When $y=\frac{B^{gl}-B^{gr}+\Lambda_{p}+D-\varphi \Psi x}{\vartheta \Gamma -\theta C^{cp}+\Lambda_{p}}=y^{*}$, for any *z*, *F*(*z*)≡0. At this time, any governmental departments' strategy is a stable strategy.

(b) If *y* ≠ y^*^, then set *F*(*z*) = 0 to obtain two stable points, *z* = 0 and *z* = 1. When *y* > y^*^, ${\frac{\partial F\left(z\right)}{\partial z}|}_{z=0}\;<\;0$, $and\;{\frac{\partial F\left(z\right)}{\partial z}|}_{z=1}>0$, then *z* = 0 is the evolutionary stable point; that is, when the proportion of domestic enterprises choosing “employee management systems” is larger than a certain degree, “loose supervision” is the optimal strategy for governmental departments. When *y* < y^*^, ${\frac{\partial F\left(z\right)}{\partial z}|}_{z=1}<0$, and ${\frac{\partial F\left(z\right)}{\partial z}|}_{z=0}>0$, then *z* = 1 is the evolutionary stable point; that is, when the proportion of domestic enterprises choosing “employee management systems” is less than a certain degree, “strict supervision” is the optimal strategy for governmental departments. The related phase diagram is shown in Figure 4.

Figure 4 shows the volume *V*_*g*1_ of the probability that governmental departments choose to implement “strict supervision” and the volume *V*_*g*2_ of the probability of choosing to implement “loose supervision,” which can be calculated by the following:


(17) $$\begin{array}{l} V_{g1}=\int_{0}^{1}\int_{0}^{\frac{B^{gl}-B^{gr}+\Lambda_{p}+D}{\varphi \Psi }}\left[\frac{B^{gl}-B^{gr}+\Lambda_{p}+D-\varphi \Psi x}{\vartheta \Gamma -\theta C^{cp}+\Lambda_{p}}\right]dxdz\\ \;=\frac{{\left(B^{gl}-B^{gr}+\Lambda_{p}\right)}^{2}}{2\varphi \Psi \left(\vartheta \Gamma -\theta C^{cp}+\Lambda_{p}\right)} \end{array}$$

(18) $$\begin{array}{l} V_{g2}=1-V_{g1} \end{array}$$

**Inference V:** The probability of governmental departments choosing “strict supervision” is positively related to the positive reputation obtained during strict supervision, punishments for the irregular behavior of domestic enterprises with employee management systems and punishments for the irregular behavior of domestic enterprises with employee management systems and is negatively related to cost differences between the strict and loose supervision of governmental departments and subsidies to clients and domestic enterprises with employee management systems.

**Proof:** Since $\frac{\partial V_{g2}}{\partial D}>0$, $\frac{\partial V_{g2}}{\partial \Lambda_{p}}>0$, $\frac{\partial V_{g2}}{\partial \theta C^{cp}}>0$, $\frac{\partial V_{g2}}{\partial \Delta B_{g}}<0$, $\;\frac{\partial V_{g2}}{\partial \varphi \Psi }<0$, $and\;\frac{\partial V_{g2}}{\partial \vartheta \Gamma }<0$, when *D*, Λ_*p*_, and θ*C*^*cp*^ increase, the volume of *V*_*g*1_ increases, and the probability of governmental departments choosing “strict supervision” increases. When Δ*B*_*g*_, φΨ, and ϑΓ decrease, the volume of *V*_*g*1_ decreases, and the probability of governmental departments choosing “strict supervision” decreases.

From Inference V, it can be seen that when governmental departments set higher punishments for the irregular behaviors of domestic enterprises or set lower subsidies for domestic enterprises and clients, it is more likely to prompt governmental departments to choose “strict supervision.” The higher the amounts of subsidies issued are, the more likely governmental departments are to choose “loose supervision.”

**Inference VI:** The probability of governmental departments choosing “strict supervision” is negatively related to the probability of clients choosing “to purchase” and is also negatively related to the probability of domestic enterprises choosing “employee management systems”.

**Proof:** When *y* < y^*^ or $x<\frac{B^{gl}-B^{gr}+\Lambda_{p}+D-\left(\vartheta \Gamma -\theta C^{cp}+\Lambda_{p}\right)y}{\varphi \Psi }$, ${\frac{\partial F\left(z\right)}{\partial z}|}_{z=1}<0$, *and F*(*z*) > 0; at this time, *z* = 1 is the evolutionary stable strategy. Therefore, as *x* or *y* decreases, the stable strategy of governmental departments increases from *z* = 0 to *z* = 1.

From Inference VI, it can be seen that if the probability of clients choosing “to purchase” from domestic enterprises or the probability of domestic enterprises choosing “employee management systems” is low, then it indicates that the market lacks benign development conditions, governmental departments are more needed to guide consumption at this time, and the profits are greater both in terms of reputation gain and punishments for the irregular behavior of domestic enterprises. Conversely, when the degree of formalization of the domestic service market is relatively high, governmental departments are more likely to choose “loose supervision”.

### 5.4. Strategic stability analysis of the co-evolution of clients, domestic enterprises and governmental departments

According to Equations (3), (9) and (15), the replicator dynamic system is as follows:


(19) $$\{\begin{array}{c} F\left(x\right)=x\left(1-x\right)\left(\varphi \Psi z+L_{ec}y-V^{eb}+V^{en}+R_{eb}-R_{en}\right)\\ F\left(y\right)=y\left(1-y\right)\left[\left(\vartheta \Gamma -\theta C^{cp}+\Lambda_{p}\right)z-L_{ec}x-Q^{ce}+Q^{cn}-W_{cn}+W_{ce}\right]\\ F\left(z\right)=z\left(1-z\right)\left[B^{gl}-B^{gr}+\Lambda_{p}+D-\varphi \Psi x-\left(\vartheta \Gamma -\theta C^{cp}+\Lambda_{p}\right)y\right] \end{array}$$

Equation (19) should be solved to obtain the equilibrium point of the systematic evolutionary game:


(20) $$\begin{array}{l} E_{1}(0,0,0),E_{2}(0,1,0),E_{3}(0,0,1),E_{4}(1,0,0),E_{5}(1,0,1),E_{6}(1,1,0),\\ E_{7}(0,1,1),E_{8}(1,1,1),E_{9}(0,\frac{B^{gl}-B^{gr}+\Lambda_{p}+D}{\vartheta \Gamma -\theta C^{cp}+\Lambda_{p}},\\ \frac{Q^{ce}-Q^{cn}-W_{ce}+W_{cn}}{\vartheta \Gamma -\theta C^{cp}+\Lambda_{p}}),E_{10}(\frac{Q^{cn}-Q^{ce}-W_{cn}+W_{ce}}{L_{ec}},\\ \frac{V^{eb}-V^{en}-R_{eb}+R_{en}}{L_{ec}},0),E_{11}(\frac{B^{gl}-B^{gr}+\Lambda_{p}+D}{\varphi \Psi },0,\\ \frac{V^{eb}-V^{en}-R_{eb}+R_{en}}{\varphi \Psi }),E_{12}(\frac{B^{gl}-B^{gr}+\Lambda_{p}+D-\vartheta \Gamma +\theta C^{cp}-\Lambda_{p}}{\varphi \Psi },\\ 1,\frac{V^{eb}-V^{en}-R_{eb}+R_{en}-L_{ec}}{\varphi \Psi }),\\ E_{13}(\frac{Q^{cn}-Q^{ce}-W_{cn}+W_{ce}-\vartheta \Gamma +\theta C^{cp}-\Lambda_{p}}{L_{ec}},\\ \frac{V^{eb}-V^{en}-R_{eb}+R_{en}-\varphi \Psi }{L_{ec}},1),E_{14}(1,\frac{B^{gl}-B^{gr}+\Lambda_{p}+D-\varphi \Psi }{\vartheta \Gamma -\theta C^{cp}+\Lambda_{p}},\\ \frac{Q^{ce}-Q^{cn}-W_{ce}+W_{cn}+L_{ec}}{\vartheta \Gamma -\theta C^{cp}+\Lambda_{p}}). \end{array}$$

In an asymmetric multigroup evolutionary game, the stability of its equilibrium point is a strict Nash equilibrium (43), which is a pure strategy equilibrium. Therefore, in Equation (19), there are only 8 equilibrium points (E_1_ to E_8_) in the dedomain *R* = {(*x, y, z*)|0 ≤ *x* ≤ 1, 0 ≤ *y* ≤ 1, 0 ≤ *z* ≤ 1}. Only E_1_ to E_8_ are studied in this paper. The local stability analysis of the Jacobian matrix of the replicator dynamic system can obtain the stability of the equilibrium point of the evolutionary system (44). According to Equation (19), the Jacobian matrix (**J**) of the system is as follows:


(21) $$\begin{array}{l} J=\left[\begin{array}{ccc} J_{1} & J_{2} & J_{3}\\ J_{4} & J_{5} & J_{6}\\ J_{7} & J_{8} & J_{9} \end{array}\right]=\left[\begin{array}{ccc} \frac{\partial F\left(x\right)}{\partial x} & \frac{\partial F\left(x\right)}{\partial y} & \frac{\partial F\left(x\right)}{\partial z}\\ \frac{\partial F\left(y\right)}{\partial x} & \frac{\partial F\left(y\right)}{\partial y} & \frac{\partial F\left(y\right)}{\partial z}\\ \frac{\partial F\left(z\right)}{\partial x} & \frac{\partial F\left(z\right)}{\partial y} & \frac{\partial F\left(z\right)}{\partial z} \end{array}\right] \end{array}$$

The eigenvalues of each equilibrium point can be obtained from the Jacobian matrix of the system. According to Lyapunov's indirect method (45), if the eigenvalues are positive or negative, then the equilibrium point is a saddle point; if the eigenvalues are all positive, then the equilibrium point is an unstable point; and if the eigenvalues are all negative, then the equilibrium point is an asymptotic stable point. This strategic combination is an evolutionary stable strategy. Accordingly, stability analysis can be carried out for each equilibrium point. Due to space limitations, the case in which λ is a positive number is not shown in Table 3.

**Table 3:** Local stability analysis for equilibrium point.

**Equilibrium points**	**λ**	**Characteristic equations**	**Symbols**
E_1_(0,0,0)	λ_1_	$V^{en}-V^{eb}+R_{eb}-R_{en}$	Uncertain
	λ_2_	$Q^{cn}-Q^{ce}-W_{cn}+W_{ce}$	Uncertain
	λ_3_	$B^{gl}-B^{gr}+\Lambda_{p}+D$	Uncertain
E_4_(1,0,0)	λ_1_	$V^{eb}-V^{en}-R_{eb}+R_{en}$	Uncertain
	λ_2_	$Q^{cn}-Q^{ce}-W_{cn}+W_{ce}-L_{ec}$	Uncertain
	λ_3_	$B^{gl}-B^{gr}+\Lambda_{p}+D-\varphi \Psi$	Uncertain
E_6_(1,1,0)	λ_1_	$V^{eb}-V^{en}-R_{eb}+R_{en}-L_{ec}$	−
	λ_2_	$L_{ec}+Q^{ce}-Q^{cn}+W_{cn}-W_{ce}$	Uncertain
	λ_3_	*B*^*gl*^ − *B*^*gr*^ + *D* − φΨ − ϑΓ	Uncertain
E_8_(1,1,1)	λ_1_	$V^{eb}-V^{en}-R_{eb}+R_{en}-\varphi \Psi$	−
	λ_2_	$\theta C^{cp}-\Lambda_{p}+L_{ec}+Q^{ce}-Q^{cn}$	−
	λ_3_	ϑΓ + φΨ − *D* − *B*^*gl*^ + *B*^*gr*^	Uncertain

E_6_ and E_8_ are the ideal combination of strategies for the regularization of the domestic service industry, and only these two situations are analyzed below.

**Inference VII**: When governmental departments choose “loose supervision,” (1, 1, 0) is the combination of evolutionary stable strategy.

**Proof:** When $L_{ec}+Q^{ce}-Q^{cn}+W_{cn}-W_{ce}<0$ and *B*^*gl*^ − *B*^*gr*^ + *D* − φΨ − ϑΓ + θ*C*^*cp*^ < 0, E_6_ is the evolutionarily stable strategy.

From Inference VII, it can be shown that this situation exists in the stage of high marketization of the domestic service industry. Even when subsidy intensity is low, clients still tend to choose to purchase services from domestic enterprises with employee management systems, and domestic enterprises are more inclined to implement “employee management systems”. Since the marketization of domestic services is the general trend of industry development, this situation is ideal for the formalization of the domestic service industry.

**Inference VIII:** When governmental departments choose “strict supervision,” (1, 1, 1) is the combination of evolutionary stable strategy.

**Proof:** When Γ + φΨ − *D* − *B*^*gl*^ + *B*^*gr*^ − θ*C*^*cp*^ < 0, *E*_8_ is the evolutionarily stable strategy.

From Inference VIII, it is shown that if governmental departments' subsidies are relatively high for domestic enterprises and clients and the profits of “strict supervision” by governmental departments are also relatively high, then governmental departments tend to guide the formalization of the domestic service industry through policies. At this time, domestic enterprises choose to implement “employee management systems” due to the increase in profits, and clients tend to choose to purchase services from domestic enterprises due to the increase in profits. Therefore, this situation is also the ideal situation for the formalization of the domestic service industry.

**Figure 5:**
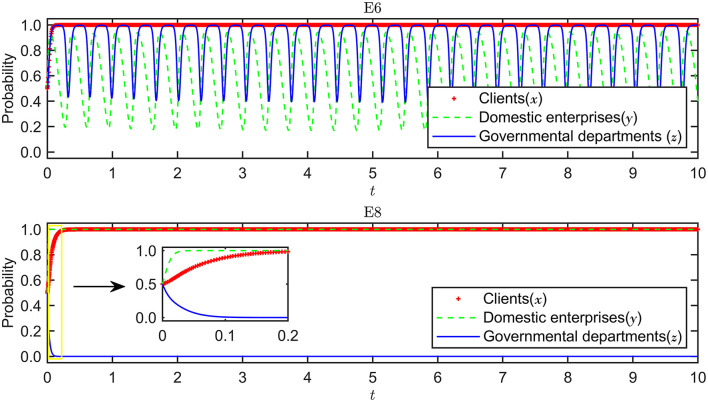
Evolutionary stability of E_6_ and E_8_.

The validity tests of the system evolution stability of E_6_ and E_8_ are shown in Figure 5, the results of which show that they can both converge to the stable situation. In the next section, we explore these two situations through systematic simulation analysis.

## 6. Simulation results

In this paper, we use Stata17 for descriptive statistics of the data and use MATLAB R2021b to simulate the dynamic evolution process of the system.

From the survey data, the distribution of types of domestic enterprises is as follows: the employee management system accounts for 1.10%, the quasi-employee management system accounts for 13.10%, the agency management system accounts for 56.41%, and the self-employed (without domestic enterprises) account for 29.39%. The proportion of clients who choose to purchase domestic services through domestic enterprises is 60.28%. Therefore, the initial ideal strategy values of *x*, *y*, and *z* are set to 0.6, 0.1, and 0.5, respectively, in this paper.

According to the research assumptions and survey data, we set two sets of parameter values to carry out the system simulation analysis for E_6_ and E_8_. The initial values of System L (E_8_) are set as follows: *B*^*gr*^ = 300, *B*^*gl*^ = 50, Γ = 130, ϑ = 1, Ψ = 50, φ = 1, $L_{ec}=32,\;R_{en}=80,\;R_{eb}=60,\;V^{en}=50,\;V^{eb}=60,\;\Lambda_{p}=70,\;W_{cn}=200,\;W_{ce}=126,\;Q^{cn}=60,\;Q^{ce}=100,\;D=400,\;\theta =1$, *and C*^*cp*^ = 80. The initial values of System P (E_6_) are set as follows: $B^{gr}=320,\;\Gamma =141,\text{ Ψ}=60,\;C^{cp}=50,\;{and\;W}_{cn}=100.\;$The remaining values are set the same as those of System L.

### 6.1. Impact on changes in the proportion of the initial ideal strategy for three actors

**Figure 6:**
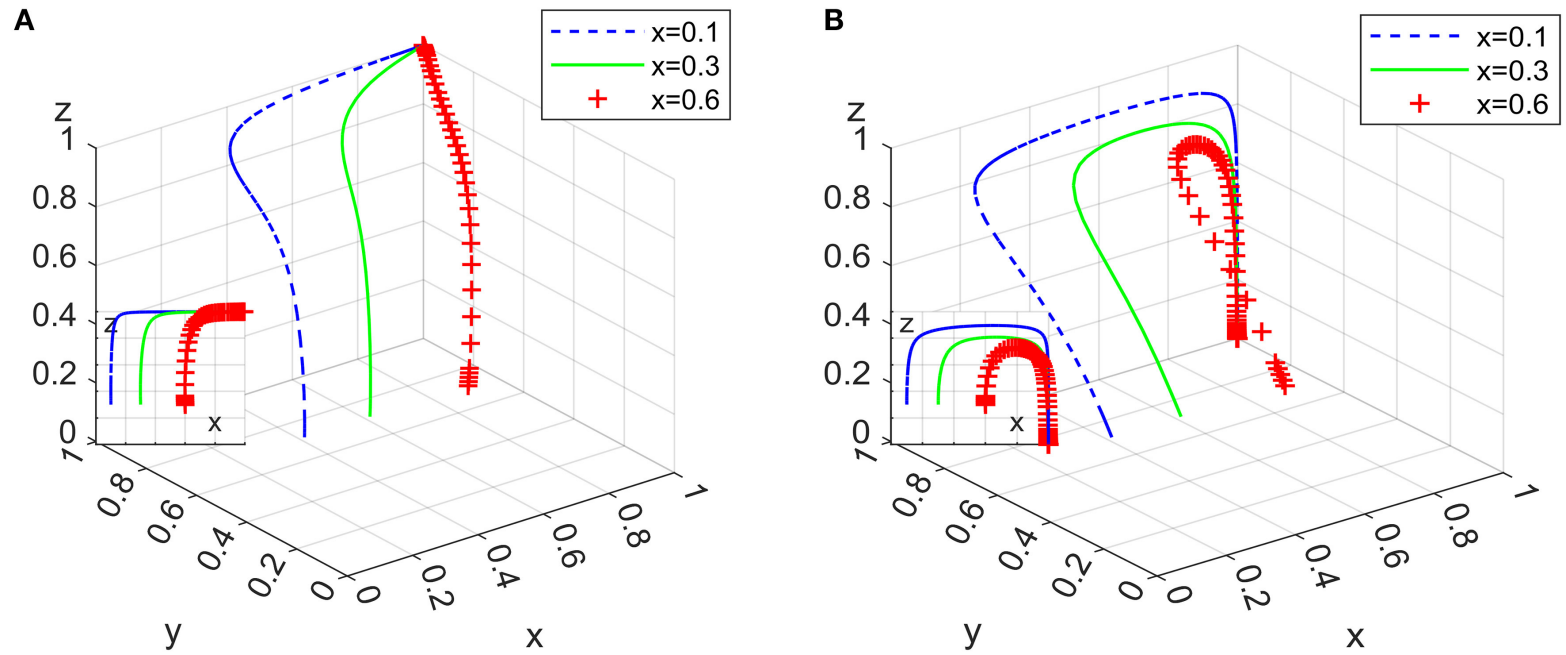
Impact of clients' strategies in system L **(A)** and system P **(B)**.

**Figure 7:**
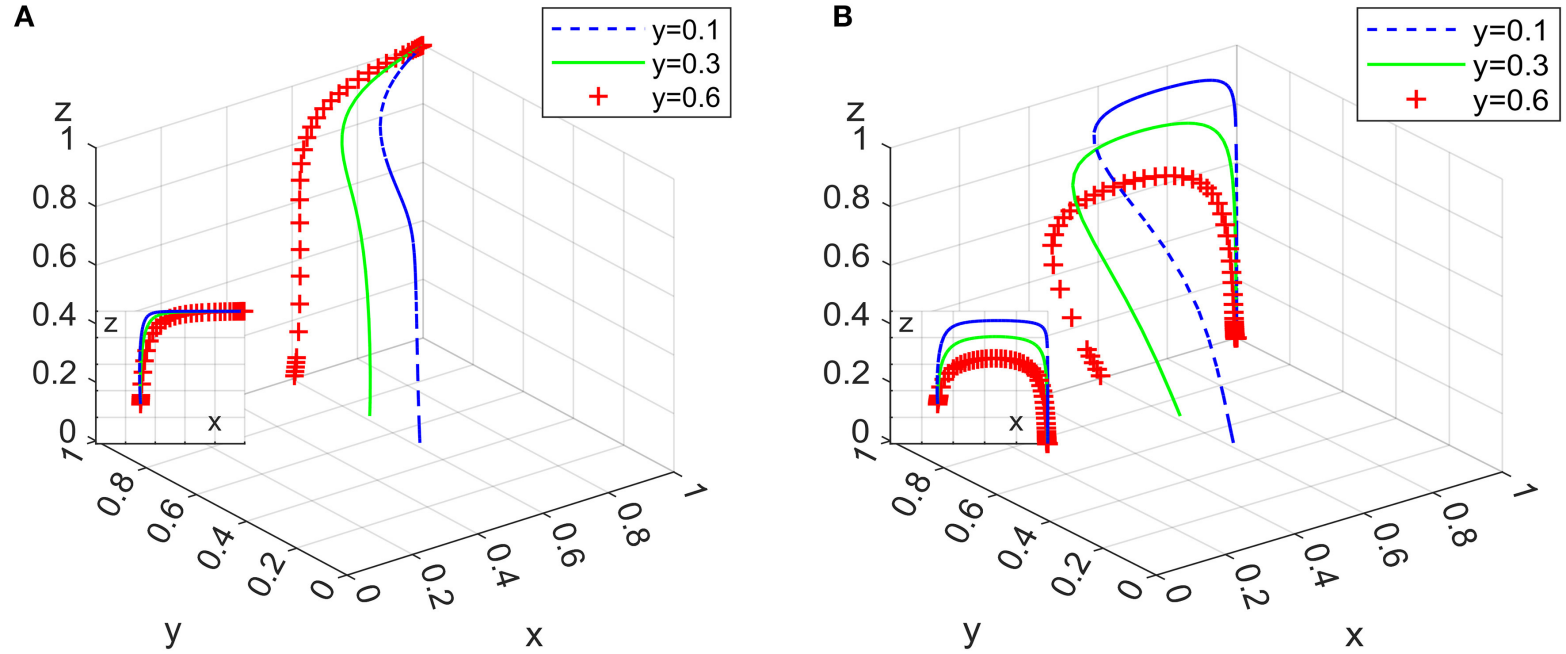
Impact of domestic enterprises' strategies in system L **(A)** and system P **(B)**.

**Figure 8:**
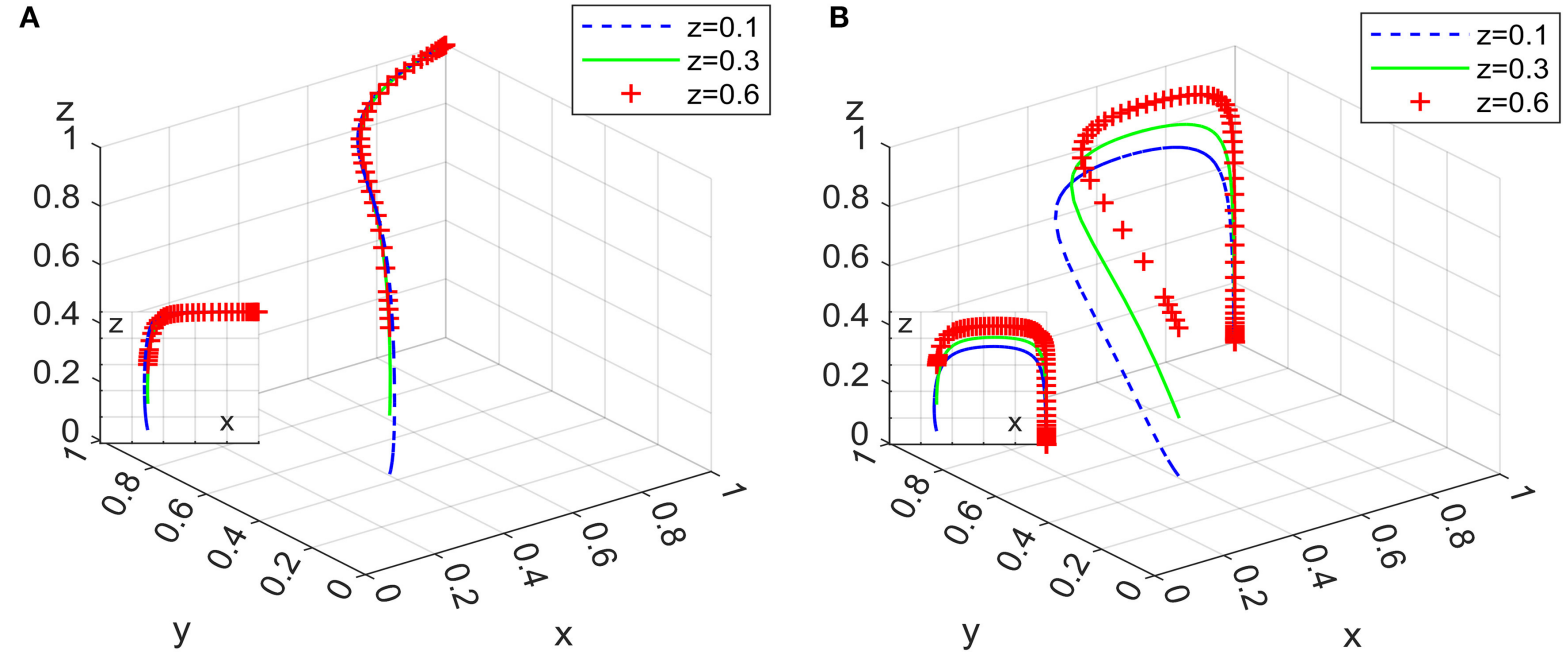
Impact of governmental departments' strategies in system L **(A)** and system P **(B)**.

Under the condition that the ideal strategy of the other two actors has a constant value of 0.3, the ideal strategy ratio of a certain actor is set to 0.1, 0.3, and 0.6, the 100 simulation results of replicator dynamic equations of System L and P in time *t* are shown in Figures 6–**8**.

Figure 6 shows that the increase in the initial proportion of clients' ideal strategy in the two systems has a significant positive impact on the system evolution process; that is, the higher the initial proportion of clients' ideal strategy is, the faster the system converges to an asymptotic stable point.

Figure 7 shows that the increase in the initial proportion of the ideal strategy of domestic enterprises in the two systems positively impacts the system evolution process. Relatively speaking, the change in *y* has a greater impact on System P than on System L. The tendency of domestic enterprises to implement employee management systems has an obvious positive effect on the formalization of the domestic service industry; in particular, governmental departments should focus on increasing the market share of domestic enterprises with employee management systems when they want to adopt loose supervision strategies.

Figure 8 shows that the initial proportion of strict supervision implemented by governmental departments in the two systems has different effects on the system evolution process. In System L, the change in *z* has no significant effect on the system converging to an asymptotically stable point. In System P, however, the increase in *z* slows down the rate at which the system converges to an asymptotic stable point, which shows that the proportion of those governmental departments implementing strict supervision should be appropriately reduced if domestic enterprises with employee management systems have a certain market share and if governmental departments implement subsidy policy as a periodic strategy.

Figures 6–8 show that in the two systems, compared with other actors in the game, the initial proportional change in clients' ideal strategy has the greatest impact on the convergence to an asymptotic stable point. This means that governmental departments' subsidies for clients are effective to speed up the system evolutionary rate.

### 6.2. Impact of governmental departments on subsidy intensity for domestic enterprises and clients

**Figure 9:**
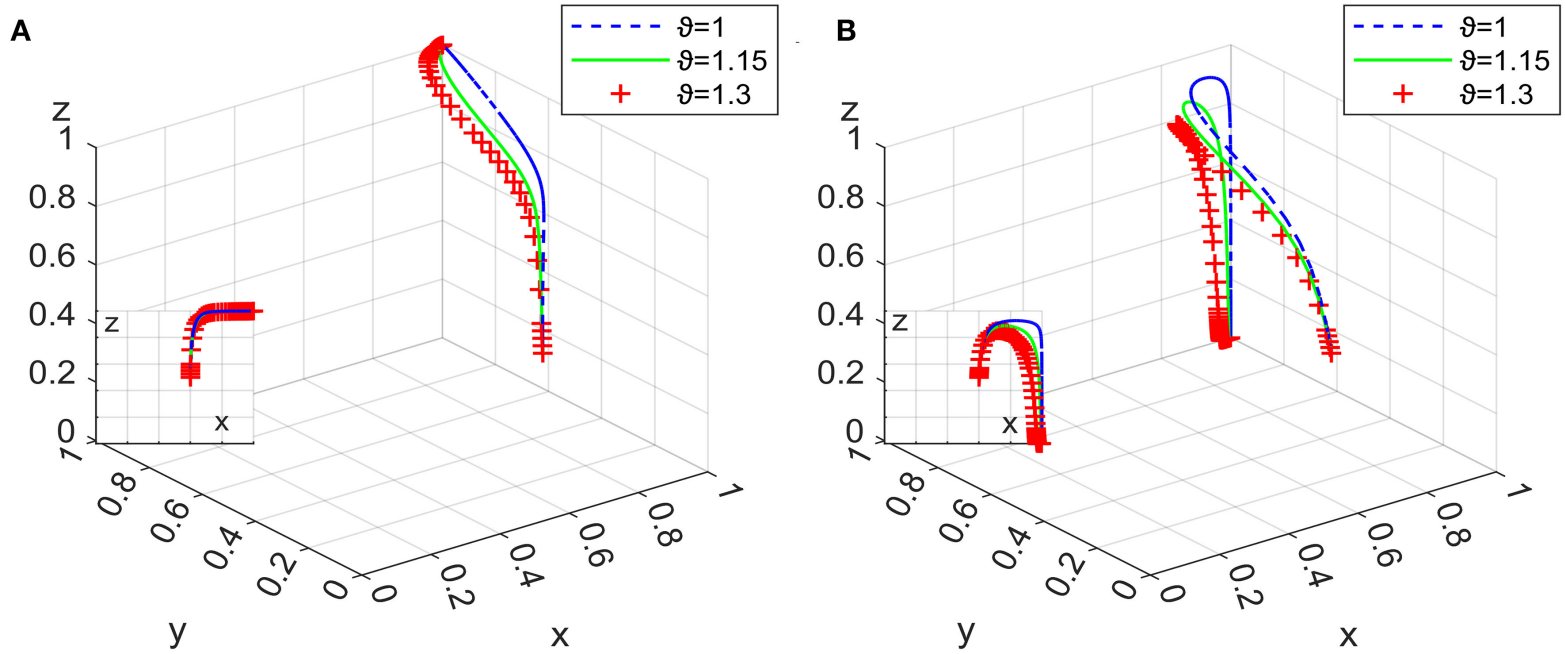
Impact on subsidy intensity for domestic enterprises in system L **(A)** and system P **(B)**.

Under the condition that other parameters remain unchanged, assigning ϑ as 1, 1.15, and 1.3, the 100 simulation results of replicator dynamic equations in System L and P in time *t*are shown in Figure 9.

Figure 9 shows that for System L, the increase in the subsidy intensity of governmental departments for domestic enterprises with employee management systems slows down the rate of its evolution to an asymptotic stable point, while for System P, it speeds up the evolutionary rate. Additionally, the increase in *y* has a greater impact on System P than on System L. Combining Inferences V and VI, it can be seen that the increase in the total cost of “strict supervision” by governmental departments reduces the probability of governmental departments adopting this strategy, thereby reducing the probability of employers purchasing services through domestic enterprises. Therefore, when governmental departments implement “strict supervision” as a long-term strategy, subsidies for domestic enterprises should be properly controlled. When the implementation of “strict supervision” is a periodic strategy for governmental departments, subsidies for domestic enterprises should be increased as appropriate to promote the formalization of the domestic service industry.

**Figure 10:**
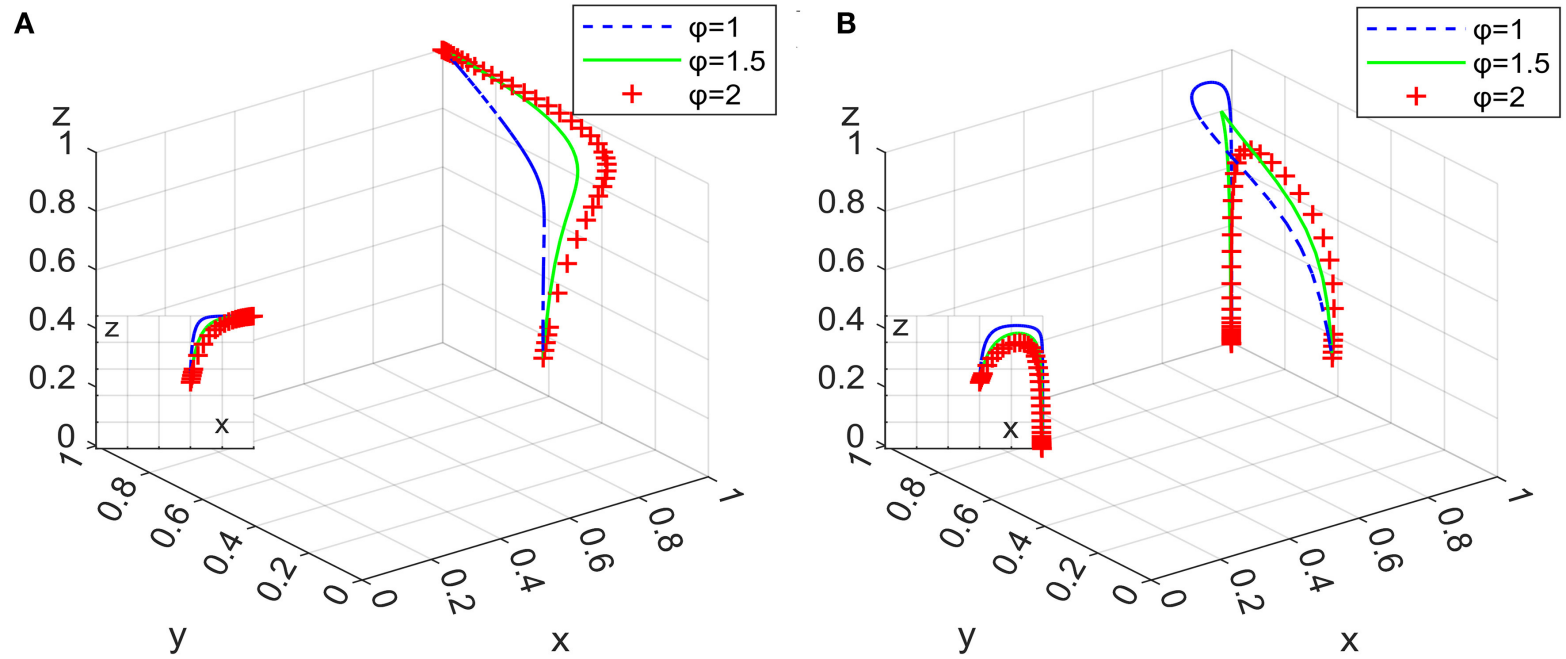
Impact on subsidy intensity for clients in system L **(A)** and system P **(B)**.

Under the condition that other parameters remain unchanged, assigning φ as 1, 1.5, and 2, the 100 simulation results of replicator dynamic equations in Systems L and P in time *t* are shown in Figure 10.

Figure 10 shows that the increase in subsidy intensity for clients in the two systems has a positive impact on the process of system evolution. Combining Inferences I and VI, it can be seen that the increase in the subsidy intensity for clients can effectively increase their purchase probability, thereby speeding up the rate at which the system converges to an asymptotically stable point, meaning that regardless of whether governmental departments take subsidy policy as a long-term or periodic strategy, they should attach importance to subsidies for clients. In addition, Figures 9, 10 show that there are different degrees of marginal diminishing effects on the increase in the amount of subsidies for domestic enterprises and clients.

### 6.3. Impact of governmental departments on punishment intensity for domestic enterprises

**Figure 11:**
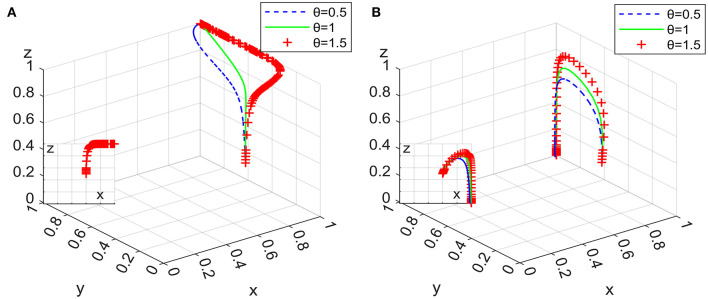
Impact on punishment intensity in system L **(A)** and system P **(B)**.

Under the condition that other parameters remain unchanged, assigning θ as 0.5, 1, and 1.5, the 100 simulation results of replicator dynamic equations in System L and System P in time *t* are shown in Figure 11.

Figure 11 shows that there are differences in the impact of increasing the punishment intensity on irregular behavior for domestic enterprises with employee management systems in terms of convergence to an asymptotic stable point in different systems, and System L is more sensitive to such changes than System P is. In the process of converging to the asymptotic stable point of System L, the positive effect of increasing punishment intensity increases first and then decreases, while in System P, it shows a decreasing trend. Combining Inferences I and III, it can be seen that increasing the punishment intensity for the irregular behavior of domestic enterprises with employee management systems reduces their market share, thereby reducing the probability of clients' ideal strategy and extending the evolutionary path of the system. Moreover, compared with the situation in which governmental departments set up exit mechanisms with supervision and subsidies, when strict supervision is implemented by governmental departments as a long-term strategy, changes in punishment intensity for domestic enterprises have a more significant impact on the evolutionary path of the system.

## 7. Conclusions and contributions

By constructing a tripartite evolutionary game model of clients, domestic enterprises and governmental departments, this study conducts a theoretical analysis of the key factors that affect the choices and co-evolutionary strategies of actors and their mechanisms and uses the field survey data of domestic workers in four cities to assign values to the evolutionary game models for simulation analysis, and strive to reveal the mechanisms for the upgrading of the domestic service industry and the optimization of the business structure in China. The formalization of the domestic service industry is beneficial to improve the quality of elderly care, further promote the positive work experience of domestic workers engaged in home-based elderly care, provide market-oriented and standardized industrial support to the elderly care model with a high proportion of home-based elderly care, and establish a compensatory social network to support this elderly care model, which rescue the urgent dilemma of Chinese elderly care significantly.

### 7.1. Conclusions

The main conclusions of this study are presented below.

First, the initial ideal strategy ratios and differences between the profits and costs of each actor, subsidies to clients, and subsidies and punishments for the irregular behavior of domestic enterprises are the key factors that cause the system to converge to a gradual stable point, reflecting the formalization of the domestic service industry. The lower the initial proportion of the ideal strategy of clients and/or domestic enterprises is, the higher the probability that governmental departments choose to implement strict supervision. In addition, the punishment of irregular behavior for domestic enterprises with employee management systems by governmental departments should be less than the setting of its subsidies, which is more conducive to the realization of the formalization of the domestic service industry.

Second, policy planning can be divided into long-term or periodic planning, and the effect of certain key factors on the evolution of the system is different in different situations. Thus, the priorities of policies under different programs should be different. When governmental departments regard the implementation of subsidies and supervision as a long-term strategic plan, they should appropriately control subsidies to domestic enterprises. The rising costs of governmental departments reduce the probability of their choice to implement strict supervision, thereby reducing the probability of clients purchasing services through domestic enterprises and delaying the realization of the formalization of the domestic service industry. When governmental departments formulate exit mechanisms with subsidies and supervision, it is more efficient to promote the formalization of the domestic service industry by focusing on increasing the proportion of domestic enterprises with employee management systems to a certain market share and gradually reducing the proportion of those implementing strict supervision.

Third, the current subsidy policy only focuses on domestic enterprises, which is, to some extent, inefficient in promoting the formalization of the domestic service industry, because the initial proportional change in clients' ideal strategy has the greatest impact on the convergence to an asymptotic stable point.

### 7.2. Policy implications

Based on research conclusions and social reality, this study puts forward the policy recommendations presented below.

First, the subsidy structure should be continuously optimized. Initially, the subsidy targets at this moment are mainly domestic enterprises and domestic workers, and the emphasis on clients should be increased. For example, governmental departments can issue consumer vouchers to households with home-based elderly care needs to reduce the purchase costs, and attract clients to purchase domestic services through domestic enterprises with employee management systems, which it could, formulate more targeted subsidy plans consequently. Compared with other kinds of domestic workers, elderly care workers generally have larger age structures and lower educational levels, and the advantages of domestic enterprises with employee management systems in providing social security and skills training may be difficult to realize. In this regard, it is possible to attract elderly care workers to choose to be employed through domestic enterprises with employee management systems by purchasing domestic accidental insurance for them. Specifically, governmental departments could expand the scope of services for the elderly by encouraging domestic enterprises with employee management systems to open community restaurants and cooperate with elderly care institutions.

Second, relevant governmental departments at all levels should adapt to local and time-sensitive conditions in the process of policy refinement and implementation. For example, domestic enterprises with employee management systems have a management model with high costs and profits. In regions with a low degree of the formalization of the domestic service market, domestic enterprises whose main business is elderly care services can be subsidized preferentially to encourage the development of a few typical enterprises, and there is no need to be vigorously promoted in the short term. As in small cities, with typical acquaintance society characteristics, households in need of elderly care services and elderly care workers are both more inclined to be introduced by familiar people to establish market relationship. In such cases, it is more difficult to popularize domestic enterprises with employee management systems. In some developed regions, policies can be more focused on exploring how to subsidize households in the need of elderly care services, accordingly with establishing policy supervision as well as evaluation mechanisms, with a principle cause that the larger the urban population, the faster the population mobility, and the stronger the population heterogeneity. In such a situation, it is easier to implement employee management systems which need higher professional standards.

Third, governmental departments should pay a great deal of attention to measures that can improve the quality of elderly care services. On the one hand, it is possible to develop domestic training materials and courses that are suitable for the current situation of home-based elderly care in Chinese society by cooperating with excellent domestic enterprises and universities to improve the professional skills and quality of elderly care workers. On the other hand, the elderly care service demand side can be included to establish long-term evaluation mechanisms to improve subsidy policy.

Fourth, executive branches cooperate with market organizations to implement evaluation and supervision programs. For example, it is possible for authorities in the field of domestic services to cooperate with relevant units, such as big data resource administrations, to establish domestic platforms that require real-name authentication. The content of the platforms should include the integrity management records of domestic enterprises, details of subsidy implementation, personal certification of domestic workers and clients, service evaluations, etc.

### 7.3. Limitations

This study also has certain limitations. First, the construction and analysis of evolutionary game models are based on relevant assumptions. Social phenomena are complex, and some important factors are abstract and difficult to represent numerically. The processing of these factors affects the process of system evolution to stable points. Although this study makes assumptions based on theory and reference to the previous literature in the process of constructing evolutionary game models, it cannot be guaranteed to be sufficiently perfect. Second, since it takes a certain period of time from the setting of subsidy policy to their implementation, the current subsidy policy has been implemented for a relatively short period of time, and there is still a lack of data on the financial expenditures of relevant governmental departments for verification purposes.

## Data availability statement

The raw data supporting the conclusions of this article will be made available by the authors, without undue reservation.

## Author contributions

All authors listed have made a substantial, direct, and intellectual contribution to the work and approved it for publication.
